# BACKWARD GAIT CHARACTERISTICS: A NOVEL PERSPECTIVE ON PHYSICAL FUNCTIONING

**DOI:** 10.1093/geroni/igae098.2805

**Published:** 2024-12-31

**Authors:** Braden Popelsky, Kelly Ylitalo

**Affiliations:** Baylor University, Bryan, Texas, United States; Baylor University, Waco, Texas, United States

## Abstract

Mobility is fundamental to healthy aging. Mobility measures such as gait velocity, double support phase, step length, and asymmetry can quantify ambulation and identify gait patterns that signify compensation for unsteadiness, poor balance, and physical functioning limitations. Forward walking is well-established as a measure of physical functioning, but fewer studies have assessed backward direction. Independently ambulatory young adults (n=20; age: 22.9 years ±1.6) and community-dwelling older adults (n=19; age: 70.2 years ±6.2) completed repeated forward and backward walking tests on a 3.9-m instrumented pressure mat. Paper surveys assessed sociodemographic characteristics, physical activity, fall history, and self-reported physical functioning (Physical Component Score (PCS) from the Short-Form Health-Survey 12). Forward and backward gait measures were evaluated using principal component analysis (PCA), and receiver-operating curves were used to model the diagnostic efficacy of gait measures on poor physical functioning. Four principal components explained 87.6% of the variance in gait. Principal component 1 accounted for 49.1% of the variance in gait with average backward step length (0.424) and backward walking speed (0.422) exhibiting the greatest loadings, indicating stronger influence. Principal component 1 was in moderate agreement (r=0.43, p=0.012) with PCS scores. Among older adults, backward gait velocity was a better predictor of poor physical functioning (PCS ≤ 50) than forward gait velocity (p=0.044; AUC=0.682 vs. 0.546). Backward gait characteristics may be novel indicators of physical functioning. Prospective studies should establish cut points for backward gait measures that classify poor physical functioning in diverse older adult populations.

